# The New "British Red Cross Society"

**Published:** 1905-07-22

**Authors:** 


### THE NEW "BRITISH RED CROSS SOCIETY."

The formation of a new Red Cross Society by their Majesties the King and Queen may be described as the chief event of the year in the field of philanthropy. The existing institutions for giving help to the sick and wounded in war have flourished exceedingly in the comparatively few years of their existence. From private institutions, originating from the effort of a few individuals they have rapidly become national and indeed international enterprises. But even now some more comprehensive scheme is still needed in the British Empire, and there is no doubt that, by amalgamating societies whose aims are almost identical, an organisation may come into existence which will be a monument for all time to the sentiments of humanity and patriotism of this generation. It is more than satisfactory, therefore, to know that the preliminary steps have already been taken towards establishing the new "British Red Cross Society." A council, appointed by their Majesties, held their first meeting on Monday at Buckingham Palace under the presidency of the Queen. The proceedings commenced with an address from her Majesty, in which she said that it had been on her mind ever since the South African war, to try and reorganise the Red Cross Society on a more practical and sound basis, and she went on to propose that

"This new organisation should be based upon membership association and the members and associates of the society shall be recruited from all classes throughout the Empire.

"The society shall be entirely voluntary, and, while in touch with the War Office and Admiralty, the society shall be organised and act wholly independently of those Departments in time of peace, but naturally in time of war it must be under naval and military control.

"I therefore now appeal to all the women of the Empire to assist me in carrying out this great scheme, which is essentially a woman's work, and which is the one and only way in which we can assist our brave and gallant Army and Navy to perform their arduous duties in time of war."

Lord Rothschild said:—

"May it please your Majesty,—I rise with great diffidence in the presence of so many illustrious persons who are intimately connected with the art of war and the requirements of the naval and military forces of his Majesty. My apology for doing so must be the fact that his Majesty the King and you, your Majesty, have kindly selected men to be the chairman of the New Red Cross Organisation which your Majesty has in such eloquent words inaugurated to-day. There may be also another reason. I am, unfortunately, one of the few survivors connected with the original Red Cross Association, which has existed in England for 35 years. When the Franco-German war broke out in 1870 that gallant soldier, the late Lord Wantage, who had planted the colours of his regiment on the heights of the Alma, for which he received the Victoria Cross, and who had served with distinction all through the campaign in the Crimea —Lord Wantage, who knew from experience the misery and sufferings of wounded and sick soldiers in a campaign, who was aware of how little was done in those days to alleviate men fighting for the honour and glory of their Sovereign and country, took advantage of the rules of the new Geneva Association to start the society over which he long presided—namely, the National Society in Aid of the Sick and Wounded in War. His Majesty the King was a patron of that society, and his Royal Highness the Duke of Connaught one of the trustees. The sympathy of the British nation for the brave soldiers and sailors of France and Germany enabled Lord Wantage at that time to collect a considerable sum of money. Speaking from memory, I think we spent about £300,000. I am sure that in no future war, whether we are engaged ourselves, which I hope may be long distant, or whether we come to the assistance of soldiers of other nations fighting, no Red Cross organisation in this country will ever attempt to do what we and other voluntary associations did on that occasion. It is fully recognised now that the care of the wounded in war, and of the sick, is as much a part of military organisation as the ordnance department, or the transport, or the commissariat, all of which are necessary for an army to be victorious in the field. Since 1870 the National Aid Society utilised the funds at their disposal and the subscriptions they received on various and numerous occasions. We rendered assistance in the Russo-Turkish war, in the Servian and Bulgarian campaigns, in the Turco-Greek war, and also the Russo Japanese war. We helped the British troops in South Africa and on various occasions in Egypt, and we offered our services to the Indian Government, but they were declined. Prior to the last South African war, Lord Lansdowne and the War Office formed a Central Red Cross Committee, On that committee the War Office was represented, St. John Ambulance Society, the Nursing Reserve Association, and the National Aid Society. We received from the public, I think, about £150,000, which we spent in South Africa, and we rendered assistance in organisation and other ways to the numerous charitable organisations which were started during the war and which rendered valuable assistance to our troops—the Yeomanry hospital, which did admirable work under the presidency of the Countess of Howe, the Duke of Portland's hospital, Lord Iveagh's Scotch and Welsh hospitals, and many others. The St. John Ambulance, under the able presidency of Lord Knutsford, sent trained bearers to the field of battle; and whilst I am speaking I hope I may be allowed to add my humble testimony to the valuable and important work which that institution is always doing in the civil life of this country. The Nursing Association sent a large number of nurses to South Africa. Your Majesty, whose thoughts are always with the sick and suffering, and whose first wish and object is to alleviate misery and to make the subjects of his Majesty happy and prosperous, takes, if possible, a greater interest in the soldiers and sailors of the King than in any of the other millions of subjects who acknowledge his sway, all of whom gratefully acknowledge the interest you take in them and the benefits you confer on them, not only by the interest you take, but by the numerous acts of charity and kindness your Majesty is always performing. This being so, it was only natural that your Majesty should wish that the Red Cross Association of the United Kingdom should be worthy of its name and of the Royal patronage which was extended to it. It was owing to that feeling that this new association owes its origin. This association will comprise all the various bodies which have hitherto acted independently. This association will have the inestimable advantage that your Majesty will preside over it. It will have the great advantage that on its Council there will be those who not only have the intimate knowledge of what sailors and soldiers require in time of war, but who also know to what extent and in what way an association of this kind can act in harmony with the naval and military authorities." Mr. Vokes, of the National Aid Society, was appointed secretary.

The Council, under the chairmanship of Lord Rothschild, then proceeded to appoint an executive committee, and a vote of thanks having been given to the Queen for so graciously consenting to preside, the meeting terminated.

